# The Potential of Immunotherapy for SMARCA4-Deficient Undifferentiated Uterine Sarcoma (SDUS)

**DOI:** 10.3390/biom14080987

**Published:** 2024-08-11

**Authors:** Xiaohong Yao, Ying He, Chaoxin Xiao, Ruihan Zhou, Chengjian Zhao, Wei Wang

**Affiliations:** 1 Department of Pathology, West China Second University Hospital, Sichuan University, Chengdu 610041, China; 2022224025528@stu.scu.edu.cn (X.Y.); heying626@scu.edu.cn (Y.H.); 2 State Key Laboratory of Biotherapy and Cancer Center, West China Hospital, Sichuan University, Chengdu 610041, China; 2022324060022@stu.scu.edu.cn (C.X.); ruihanzhou@stu.scu.edu.cn (R.Z.); 3 Key Laboratory of Birth Defects and Related Diseases of Women and Children, Sichuan University, Ministry of Education, Chengdu 610041, China

**Keywords:** SMARCA4, SMARCA4-deficient undifferentiated uterine sarcoma, endometrial stromal sarcomas, multiplex immunofluorescence, tumor immune microenvironment, immunotherapy, immune cell interactions, cellular niches

## Abstract

(1) Background: SMARCA4-deficient undifferentiated uterine sarcoma (SDUS) is a rare and aggressive cancer that urgently requires novel therapeutic strategies. Despite the proven efficacy of immunotherapy in various cancer types, its application in SDUS remains largely unexplored. This study aims to investigate the immune microenvironment of SDUS to evaluate the feasibility of utilizing immunotherapy. (2) Methods: Multiplex immunofluorescence (mIF) was employed to examine the immune microenvironment in two cases of SDUS in comparison to other subtypes of endometrial stromal sarcomas (ESSs). This research involved a comprehensive evaluation of immune cell infiltration, cellular interactions, and spatial organization within the tumor immune microenvironment (TiME). Statistical analysis was performed to assess differences in immune cell densities and interactions between SDUS and other ESSs. (3) Results: SDUS exhibited a significantly higher density of cytotoxic T lymphocytes (CTLs), T helper (Th) cells, B cells, and macrophages compared to other ESSs. Notable cellular interactions included Th–CTL and Th–B cell interactions, which were more prominent in SDUS. The spatial analysis revealed distinct immune niches characterized by lymphocyte aggregation and a vascular-rich environment, suggesting an active and engaged immune microenvironment in SDUS. (4) Conclusions: The results suggest that SDUS exhibits a highly immunogenic TiME, characterized by substantial lymphocyte infiltration and dynamic cellular interactions. These findings highlight the potential of immunotherapy as an effective treatment approach for SDUS. However, given the small number of samples evaluated, these conclusions should be drawn with caution. This study underscores the importance of additional investigation into immune-targeted therapies for this challenging cancer subtype, with a larger sample size to validate and expand upon these preliminary findings.

## 1. Introduction

Endometrial stromal sarcoma (ESS) is a rare malignant tumor originating in the uterus, which can be classified into low-grade endometrial stromal sarcoma (LG-ESS), high-grade endometrial stromal sarcoma (HG-ESS), and undifferentiated uterine sarcoma (UUS) [1]. The standard treatment for uterine stromal sarcoma is total hysterectomy and bilateral salpingooophorectomy (BSO). The five-year survival rate for uterine sarcomas ranges from 41% to 95%, depending on the specific subtype of sarcoma and the extent of metastasis [2].

SMARCA4-deficient tumors are a group of aggressive neoplasms characterized by the inactivation of the SMARCA4 gene, a key component of the SWI/SNF chromatin remodeling complex. These tumors can manifest in various organs, such as thoracic sarcomas, pulmonary adenocarcinomas, and undifferentiated carcinomas, showing rapid disease progression and poor prognosis. SMARCA4-deficient uterine sarcoma (SDUS) is a monogenic cancer distinguished by its rapid invasiveness resulting from the inactivation of the SMARCA4 gene [3]. Individuals afflicted with this subtype commonly manifest at a younger age and undergo swift disease advancement, culminating in a median survival rate of roughly seven months. Presently, there is no efficacious treatment for this condition [4]. Prior investigations have suggested that tumors deficient in the SMARCA4 gene may display increased susceptibility to immunotherapy, offering the prospect of enhanced clinical results [5].

Cancer immunotherapy operates through the activation of the immune system to specifically attack and eliminate malignant cells, leading to the eradication of tumors. This strategy is distinguished by its minimal occurrence of adverse effects and capacity to generate memory cells, thus displaying enduring and extensive therapeutic outcomes across diverse tumor categories [6]. In the field of cancer investigation and medical practice, immune checkpoint inhibitors (ICIs) have surfaced as a swiftly advancing domain of focus in cancer therapy [7]. Although there have been notable achievements, additional endeavors are necessary to pinpoint appropriate candidates for immune-based therapeutics. A comprehensive understanding of the tumor immune microenvironment (TiME) is crucial for efficiently selecting patients, developing combination treatment plans, and determining sequencing strategies [8]. TiME consists of a wide range of cellular components involved in immune-mediated cytotoxicity and immune-regulatory activities. The composition, distribution, and interactions of immune cells within the TiME collectively shape the immunological landscape of individual tumors, thereby exerting a substantial impact on the efficacy of immunotherapeutic strategies [9,10]. The complex spatial arrangement of diverse immune cell subsets and their crosstalk with tumor-infiltrating immune cells in brain tumors, breast cancer, lung adenocarcinoma, melanoma, and sarcoma is intricately connected to cellular phenotypes, functions, and clinical outcomes [11,12,13,14,15]. Therefore, investigating the spatial arrangement of the TiME in uterine sarcoma could provide important insights into the interaction between tumors and the immune system, as well as identify potential prognostic markers for the effectiveness of immune therapy. In this research, we utilize multiplex immunofluorescence (mIF) staining combined with quantitative digital image analysis to assess the spatial organization of various cellular elements in ESS.

## 2. Materials and Methods

### 2.1. Patients and Samples

At West China Second University Hospital, Sichuan University, a total of 4 cases of HG-ESS, 3 cases of LG-ESS, and 2 cases of SDUS were identified. Tissue samples were obtained from paraffin-embedded specimens collected during routine clinical diagnostics in the Department of Pathology. Among the cases, two instances of SDUS demonstrated a loss of Brg-1 expression on immunohistochemistry, which was subsequently confirmed through next-generation sequencing (NGS) to detect mutant expression of the SMARCA4 gene. Clinical and pathological data, treatment details, and post-treatment information were retrieved from electronic health records. Survival outcomes were evaluated from the time of diagnosis until the date of death, with patients who were still alive being censored at their last follow-up (Supplementary Table S1).

### 2.2. Multiplex Immunofluorescence (mIF)

Immunostaining was conducted to evaluate immune cell markers (CD3, CD4, CD8, CD14, CD20, and CD68), immune checkpoints (PD-1 and PD-L1), blood vessel markers (CD31), smooth muscle markers (α-SMA), fibroblast activation markers (FAP—fibroblast activation protein), markers of proliferation activity (Ki67), and cytotoxicity markers (Granzyme B). The nuclei were highlighted using DAPI staining. The Supplementary Materials include a detailed methodology, while Table S2 presents data related to antibodies.

### 2.3. Tissue Imaging, Phenotyping, and Image Analysis

A component TIF image tile was exported following two rounds of image fusion utilizing ImageJ. Subsequent to importing component TIF image tiles, cell segmentation and nuclear detection were conducted using QuPath version 0.4.3. Cell classification was achieved through marker intensity and the colocalization of multiple antibodies. Cells were further categorized as CD68+ macrophages, CD3+ T cells, CD8+ cytotoxic T lymphocytes, CD4+ T helper cells, CD20+ B cells, CD14+ myeloid cells, PD-1/PD-L1+ cells, and Ki67+ proliferated cells. Additional markers present in this study included the cytotoxic protein Granzyme B, fibroblast activation marker (FAP), blood vessel marker (CD31), and smooth muscle marker (α-SMA). “Other cell types” were classified as those lacking the expression of these markers. The investigation of cellular interactions within the tumor microenvironment (TiME) involved the creation of a spatial network based on proximity, with cells within a 15 μm radius deemed potential interacting partners. This methodology enabled the identification and characterization of local cellular interactions, revealing potential functional associations. Niche profiles were constructed by quantifying the spatial distribution of different cell types surrounding individual reference cells. Subsequently, clustering algorithms, such as K-means, were utilized to categorize these niches into discrete clusters, known as C-niches, based on the similarity of their cellular compositions [16].

## 3. Results

### 3.1. Comparative Analysis of Immune Infiltration in ESS vs. SDUS

**Figure 1:**
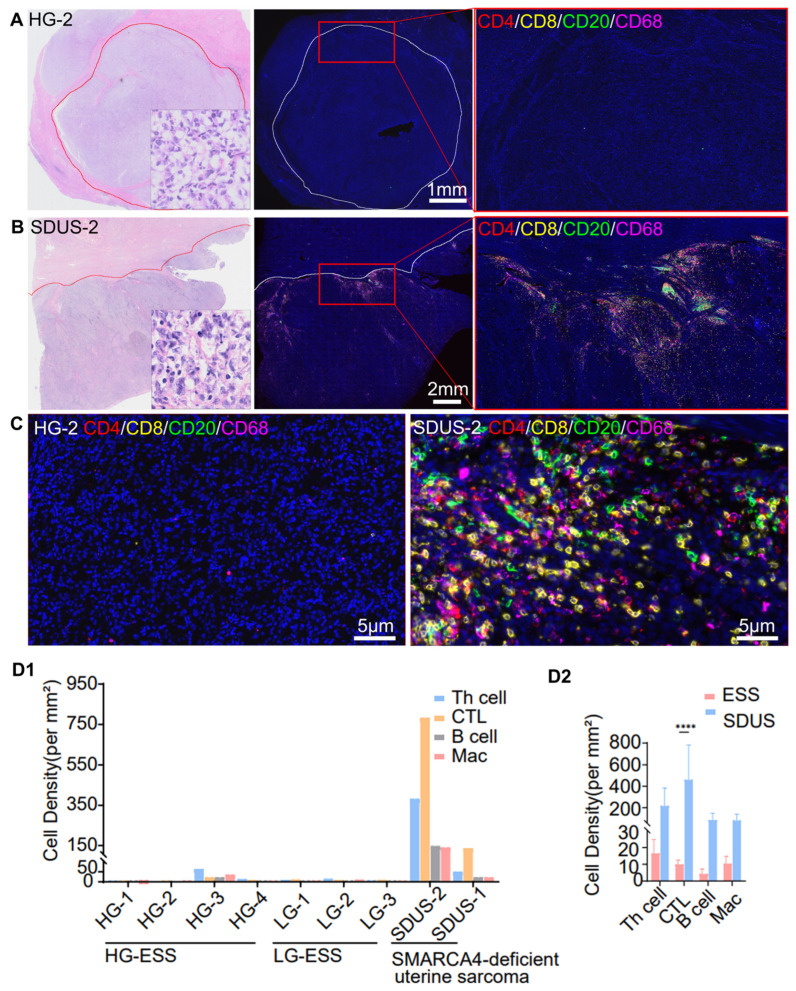
mIF defines the TiME of ESS. (**A**) A schematic diagram depicting mIF-multiplexed images from 9 patients with ESS. The images were created with FigDraw. Immune cells were identified using fluorescent dyes: CD8 (yellow), CD4 (red), CD20 (green), and CD68 (magenta), while nuclei were counterstained with DAPI, appearing as dark blue. (**B**) A schematic representation of high immune infiltration TiME characteristic of SDUS. (**C**) A schematic representation of low immune infiltration TiME commonly found in both LG-ESS and HG-SS. (**D1**) Comparison of cell densities (per mm^2^) for four markers (Th cell, CTL, B cell, and Mac) across various ESSs and SDUS. (**D2**) The comparative statistical analysis graph illustrates the distinctions in immune cell infiltration between ESSs with low immune cell infiltration and SDUS with high immune cell infiltration. A two-way ANOVA with multiple comparison test was used for statistical analysis by GraphPad Prism10.1.2.

To evaluate the cellular composition and spatial arrangement of the TiME in ESS, a panel of common immune cell markers (CD4, CD8, CD20, and CD68) was utilized. Antibodies were validated through monochromatic staining in tumor samples to ensure accurate staining patterns. Whole slide images were captured from four HG-ESS, three LG-ESS, and two SDUS samples. The spatial distribution of immune cell infiltration across the entire slide exhibited significant heterogeneity. Performing a cell density analysis that does not account for spatial distribution throughout the entirety of the slide may result in significant inaccuracies. Therefore, we chose to concentrate on areas with the greatest cell density for statistical examination. Patients diagnosed with LG-ESS and HG-ESS displayed a tumor profile characterized by diminished immune cell densities, particularly low T helper (Th) cell densities (median at 10.4 per mm^2^; range (0–64.5)), low CTL (cytotoxic T lymphocytes) densities (median at 9.8 per mm^2^; range (2.0–22.2)), low B cell densities (median at 1.2 per mm^2^; range (0.4–21.6)), and low macrophage densities (median at 6.6 per mm^2^; range (2.4–35.5)). Conversely, the two cases of SDUS exhibited elevated immune cell densities and clustering. SDUS-Case2 exhibited significant lymphocytic infiltration, characterized by a Th cell density of 382.0 mm^2^, a CTL density of 782.2 mm^2^, a B cell density of 147.9 mm^2^, and a macrophage density of 140.0 mm^2^. Additionally, our findings indicated a statistically significant increase in CTL levels in SDUS compared to other ESSs, with a *p*-value of less than 0.0001 (Figure 1).

### 3.2. A Spatial Atlas of the SDUS TiME: A Comprehensive Analysis

#### Characterization of SDUS Immune Infiltration

**Figure 2:**
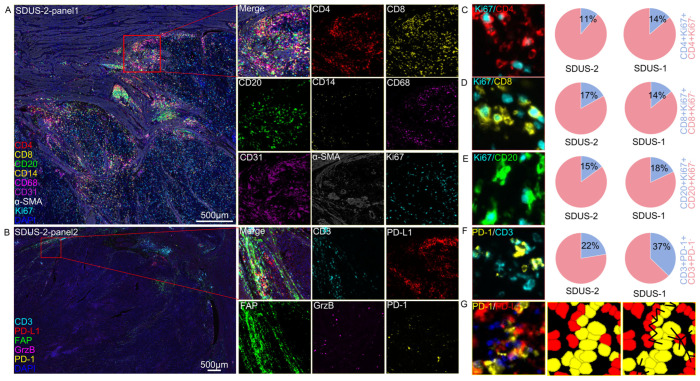
An immunofluorescent staining schematic diagram of SDUS. (**A**,**B**) The immunofluorescent staining included markers for lymphocytes (CD3, CD4, CD8, and CD20), myeloid cells (CD68 and CD14), immune checkpoint inhibitors (PD-1 and PD-L1), stromal markers (α-SMA, CD31, and FAP), and markers for cell cytotoxicity and proliferation (Ki67 and Granzyme B). (**C**–**G**) Fluorescence imaging of proliferating and exhausted cells, with a graphical representation of the proportion of proliferative cells.

We have created an extensive spatial atlas of the TiME two SDUS, analyzing and quantifying the interactions among various immune cells. In both patients, there was a notable presence of B cells, Th cells, CTLs, and macrophages, as well as angiogenesis. In SDUS-Case1, there was a reduction in the expression of proliferation markers (Ki67+) and cytotoxicity markers (Granzyme B). In SDUS-Case2, approximately 20% of the proliferating cells were CD8+ T cells, while CD4+ T cells and CD20+ B cells made up nearly 14% of the proliferating cells. In both cases, there was a presence of PD-1+ CD3+ T cells at the invasive margins of the tumor, where they were observed to interact with PD-L1+ tumor cells (Figure 2).

### 3.3. Exploring Cellular Interactions in the SDUS TiME

**Figure 3:**
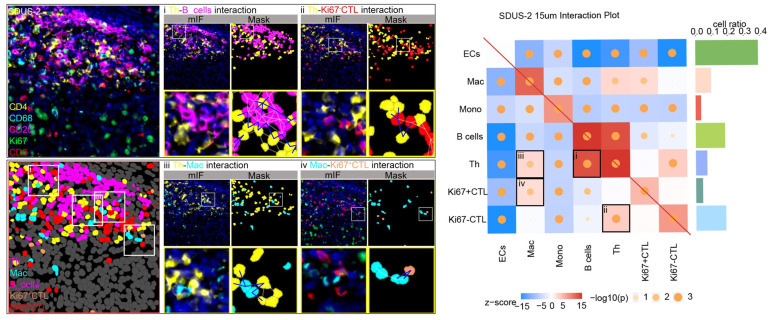
Interaction analysis of SDUS. A mIF heatmap of cell–cell interactions in patient SDUS-case2, with fluorescence images and rendering of interacting cells. Corresponding images for another patient (SDUS-case1) are provided in Supplementary Figure S1.

To gain a deeper insight into the cellular composition and spatial organization of the SDUS TiME, we conducted a comprehensive analysis of direct cell interactions and cellular niches, quantifying the spatial relationships between individual cells. Our analysis revealed Th–B cell and CTL–Th cell interactions in both cases. Case 1 exhibited unique interactions among CTL–B and CTL–Ki67+ CTLs, while Case 2 showed additional interactions between Mac–Th and Mac–Ki67+ CTLs (Figure 3).

### 3.4. Unveiling the Intricate Cellular Niches within SDUS

**Figure 4:**
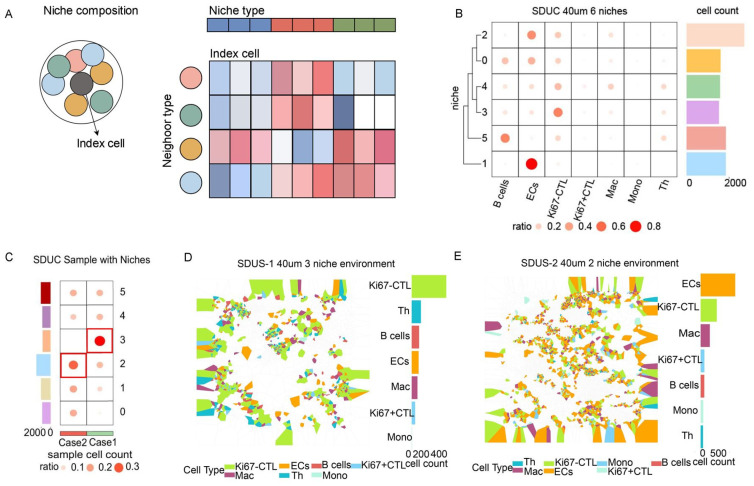
A comprehensive analysis of cellular niches in the SDUS TiME. (**A**) A niche schematic diagram [16] (**B**,**C**) presenting heatmaps displaying the cellular composition percentages and proportional distribution of niches. (**D**,**E**) depict schematic representations of the two most prevalent niches.

Within the TiME, cellular niches are distinct structures that significantly influence tumor behavior, treatment responses, and immune surveillance. In the two cases of SDUS patients studied, we observed six distinct niches, with niche 3 being the predominant niche in Case 1. Niche 3 is distinguished by the aggregation of lymphocytes, specifically CTLs, Th cells, and B cells, as well as the formation of blood vessels, with CTLs being the predominant cell type in this niche. In Case 2, niche 2 demonstrates the highest proportion, marked by a vascular-rich setting with significant angiogenesis and infiltration of CTLs and macrophages (Figure 4).

## 4. Discussion

ESSs are rare and aggressive mesenchymal tumors that currently lack effective therapeutic options. The immune microenvironment of sarcomas is intricate and dynamic. Multiple anti-tumor medications are designed to either target tumor cells or influence the TME, with a specific focus on immune cells. Additionally, immune cells are recognized as a viable treatment option for soft tissue sarcomas [15]. A comprehensive comprehension of the TiME in ESS is crucial for the advancement of innovative therapeutic strategies. This study represents the first thorough examination of the immune landscape of SDUS, incorporating analyses of cellular composition, interactions, and cell niche. The findings from these investigations have unveiled unexpected discoveries.

In our research, we utilized a multiplex immunohistochemistry staining panel to evaluate the immune microenvironment in LG-ESS, HG-ESS, and SDUS. The panel focused on assessing the presence of three main types of adaptive immune cells: Th cells, CTLs, B cells, and macrophages, which play crucial roles in the immune response to tumors [15]. CTLs are known for their strong anti-tumor capabilities, while Th cells support CTLs and B cells and can directly target tumor cells by releasing IFN-γ and TNF-α [17]. B cells, important for humoral immunity, contribute to anti-tumor responses through antibody-dependent cellular cytotoxicity and complement activation [18]. Macrophages, identified by the CD68 marker, demonstrate a significant level of plasticity, manifesting both pro- and anti-tumorigenic capabilities [19]. Our research indicates that both low-grade LG-ESS and HG-ESS exhibit an immune-low microenvironment, suggesting a state of immunosuppression possibly influenced by the genetic mutations and oncogenic pathways specific to these tumors [20]. In contrast, SDUS cases, characterized by the loss of SMARCA4, demonstrate an immune-high microenvironment, marked by substantial infiltration of tumor-infiltrating lymphocytes (TILs) and increased PD-L1 expression, indicating a potentially more immunogenic tumor that could be more responsive to immunotherapy [21]. Our statistical analysis further identified a significant disparity in the presence of CTLs between the two groups, with SDUS cases showing higher levels of CTL infiltration at the tumor invasive margin. CTLs, a crucial component of the TiME, play a pivotal role in antigen recognition, binding, and killing tumor cells expressing neoantigens. Direct interaction between tumor cells and CTLs results in tumor cell destruction. In colorectal cancer, a higher ratio of CTLs in contact with tumor cells is linked to a more favorable prognosis [22,23,24]. Elevated CD8A gene expression and increased infiltration of CTLs in triple-negative breast cancer (TNBC) contribute to enhanced antitumor immunity and improved prognosis [25]. This distinct immune environment underscores the significance of comprehending the unique immune context of sarcomas for prognostic prediction and customization of immunotherapeutic approaches [26].

The utilization of mIF staining allows for the visualization of spatial characteristics within the TiME at a single-cell resolution. Employing mIF to examine the TiME in SDUS reveals a significant presence of tumor-suppressive immune cells, including effector T cells (CTLs and Th cells), B cells, and tumor-associated macrophages [27]. Recent advancements in research suggest that immune infiltration serves as an independent prognostic biomarker and can also predict the effectiveness of conventional chemotherapy and immunotherapy based on the pre-treatment immune response [28]. Additionally, the heightened expression of Ki67 in immune cells suggests a state of proliferation, indicative of a dynamic immune microenvironment [29]. This phenomenon has the potential to bolster immune surveillance and eliminate tumor cells, ultimately leading to improved patient outcomes. Moreover, this active immune state may render the tumor more vulnerable to immunotherapeutic interventions, thereby enhancing treatment efficacy [30,31]. Furthermore, within the invasive margins of SDUS, tumor-associated T cells have been observed to express PD-1 and engage with tumor cells expressing PD-L1. The presence of the inhibitory receptor PD-1 serves as an indicator of immune activation and exhaustion within T cells. In the context of melanoma, the efficacy of immunotherapy is linked to the co-localization of PD-1-expressing T cells at the tumor periphery and PD-L1-expressing tumor cells [32]. A dynamic tumor immune microenvironment is correlated with the presence of PD-1+-activated effector T cells and the expression of PD-L1 by tumor cells. Treatment with anti-PD-1 therapy has the potential to reverse immune suppression caused by activation of the PD-1/PD-L1 axis, leading to reactivation of pre-existing tumor-specific T cells and triggering responses that result in tumor rejection [33] (Figure 2).

A complex communication network has developed among various cell types within the immune system. The interactions between cells in the immune system play a vital role in potentially improving the immune response through ICB. Analysis of the TiME landscape and interactions in two cases of SDUS revealed an accumulation of interactions between B cells and Th cells, as well as CTLs and Th cells. Th cells play a critical role in initiating effective B cell responses and producing high-affinity antibodies [34]. Moreover, B cells play an active role in cellular immune responses by influencing the magnitude and quality of T cell responses to foreign and self-antigens rather than simply receiving help from T cells [35]. Additionally, B cells also promote the activation of T cells to secrete cytokines and facilitate the development of memory lymphocytes, consequently enhancing the anti-tumor properties of T–B cell interactions [36]. Furthermore, in line with prior research [37], our study highlights the critical function of Th cells in regulating CTL responses. Th cells play a crucial role in providing necessary survival signals to CTLs, thereby diminishing activation-induced cell death (AICD) and amplifying their cytolytic activity against tumor cells. This interaction is not mediated by soluble factors but rather necessitates direct cell-to-cell contact, underscoring the significance of physical proximity between Th cells and CTLs in the TME. Moreover, the presence of Th cells not only enhances the survival of CTLs and their ability to destroy tumor cells expressing antigens, as demonstrated by heightened lytic activity in the presence of Th cells. The interaction between CTLs and Th cells is essential for an efficient immune response against tumors. Activation of antigen-specific Th cells is essential for optimal tumor immunity, underscoring the critical role of Th cell support in maintaining strong CTL function. This is in agreement with the proposed model suggesting that the expansion and tumor-targeting capacity of human cytotoxic T lymphocytes (CTLs) can be enhanced by CD4+ T-helper cells through costimulatory signals transmitted via surface molecules on the cells, leading to increased proliferation and viability of the CTLs during the effector stage of anti-tumor immune responses [38]. Consequently, there is a call for further exploration of the Th cell-CTL interaction as a promising therapeutic target to improve the effectiveness of cancer immunotherapies.

Additionally, our research identified two primary niches within the tumor immune microenvironment of SDUS: the lymphocyte aggregation niche (referred to as niche 3) and the vascular-rich niche (referred to as niche 2). The dispersion of lymphocytes throughout the tumor may impede their capacity to efficiently engage with tumor cells. Nonetheless, previous studies have demonstrated that the coordinated interaction among B cells, Th cells, and CTL within a unified immune network can effectively target and eliminate tumor cells [39]. Upon the recognition of antigens presented by antigen-presenting cells, T helper cells secrete cytokines such as IL-4, IL-5, and IL-21, which promote the proliferation, differentiation, and antibody production of B cells [40]. Furthermore, B cells have the capacity to act as antigen-presenting cells by presenting captured antigens to Th cells, thereby augmenting Th cells activation. The cytokines generated by Th cells, including IL-2, can induce the proliferation and activation of CTLs. Moreover, antibodies produced by B cells have the capability to bind to antigens present on the surface of tumor cells, creating antigen–antibody complexes that can act as indicators for CTLs to identify and combat [41]. The extensive vascular system present within the tumor is crucial in supplying vital nutrients and oxygen required to sustain the metabolic needs of immune cells that have infiltrated the tumor. Endothelial cells of the tumor’s blood vessels express chemokines and adhesion molecules that facilitate the recruitment of macrophages and CTLs to the TiME [42]. Macrophages exhibit the ability to engage in antigen presentation and cytokine secretion, leading to the activation of CTLs and thereby augmenting the anti-tumor immune response. The co-localization of macrophages and CTLs in proximity to tumor vasculature is of significant relevance in the context of treatment, as it indicates the presence of a tumor-specific immune response and offers promising targets for immunotherapy and anti-angiogenic interventions. Strategies involving the activation of CTLs through immune checkpoint inhibitors, reprogramming of macrophages, and inhibition of tumor vasculature formation have the potential to enhance the efficacy of targeted tumor therapies [43].

ICIs have shown promise as a therapeutic approach in SMARCA-altered cancers such as SMARCA4-deficient thoracic sarcoma, SMARCA4-altered non-small-cell lung cancer (NSCLC), and small cell carcinoma of the ovary, hypercalcemic type (SCCOHT) [21,44,45]. Given these findings, individuals with SDUS may be considered a potentially favorable candidate population for immunotherapy. The substantial presence of immune cells and the active immune microenvironment suggest that therapeutic interventions aimed at enhancing the anti-tumor immune response through targeting cytotoxic T lymphocytes, macrophage reprograming, and angiogenesis inhibition may be beneficial. The use of immune checkpoint inhibitors, particularly those targeting PD-1/PD-L1, could potentially overcome immunosuppression and unleash the anti-tumor potential of immune cells in the TME.

However, this study’s limited sample size, especially with only two samples representing the SDUS category, is a notable constraint due to the rarity of SDUS. Another limitation is the inability to procure tissue from patients who have received immunotherapy with ICIs. Therefore, conclusions should be drawn with caution. Nevertheless, the thorough examination of the immune microenvironment in SDUS provides a rationale for considering these individuals as suitable candidates for immunotherapeutic interventions, thus establishing a foundation for novel and improved treatment approaches in SDUS management.

## Data Availability

The data supporting the findings of this study are available within the article and its Supplementary Materials. All relevant data generated and analyzed during this study are included in this article. No new datasets were created or analyzed during the current study.

## Supplementary Materials

The following supporting information can be downloaded at: https://www.mdpi.com/article/10.3390/biom14080987/s1, Figure S1. mIF heatmap of cell–cell interactions in patient SDUS-case2, with fluorescence images and rendering of interacting cells; Table S1. Clinicopathological and molecular characteristics of ESS. Table S2. Antibody Species origin, clones and dilutions used for mIF.
