# Supplementary material for: The Potential of Immunotherapy for SMARCA4-Deficient Undifferentiated Uterine Sarcoma (SDUS)
Source: Biomolecules. 2024 Aug 11;14(8):987. doi: 10.3390/biom14080987 (PMC11352696; doi:10.3390/biom14080987)
Supplement: Supplementary file 1 [file biomolecules-14-00987-s001.zip › biomolecules-3124727-supplementary.pdf]

**Patients**	**Age(years)**	**Tumor size(cm)**	**Presenting symptoms**	**Treatment**	**Outcomes**	**LN**	**Ovary**	**Molecular Testing**
HG-1	49	Unk	Pelvic space-occupyin	LO+LND+CT	ESS recurrence	Neg	Pos	PHF1,JAZF1 Translocation
HG-2	45	6	Abnormal menstrual bleeding	TLH+BSO+LND	NA	Neg	Neg	PHF1 Translocation
HG-3	47	2.6	Abdominal discomfort	TLH+BSO+LND+CT	Disease free survival（Follow-up 10 months）	Neg	Neg	NA
HG-4	27	2.8	Prolonged menstrual cycles	TLH+LSO+CT	Disease free survival（Follow-up 10 months）	Neg	Neg	YWHAE Translocation

LG-1	61	3.7	Postmenopausal Bleeding	RSO+TCE	Disease free survival （Follow-up 12 months）	NA	Neg	YWHAE Translocation
LG-2	39	2.5	Uterine mass with Menorrhagia	TLH+BSO	Disease free survival （Follow-up 4 months）	NA	Neg	BCOR,YWHAE,TAZF1 Translocation
LG-3	39	7	Abdomina mass	TLH+BO+LND+CT+RT	Disease free survival （Follow-up 8 months）	Pos	Pos	YWHAE Translocation

SDUS-Case 1	46	17.5	Uterine enlargement abnormal uterine bleeding	TLH BSO+CT	Died due to postoperative intestinal obstruction (Time to death 7 months)	Pos	Pos	SMARCA4 mutation c.2425delA(p.I809Sfs*22)
SDUS-Case 2	58	5	Abnormal postmenopausal bleeding	TLH BSO+CT	Disease free survival （Follow-up 13 months）	Neg	Neg	SMARCA4 mutation Copy number variation deletions

Pos positive; Neg negative;NA not available； Unk unknow;TLH BSO total laparoscopic hysterectomy and bilateral salpingo-oophorectomy;CT chemotherapy;RT Radiotherapy;BO Bilateral oophorectomy;LO Left oophorectomy;LND Lymph node dissection;RSO Right salpingo-oophorectomy;TCE Tumor cell eradication;LSO Left Salpingo-oophorectomy;

Table S1. Clinicopathological and molecular characteristics of ESS

Multiplex immunofluorescence staining specific steps

To reduce interference with previously stained antibodies , TSA complexes, or epitope each antibody was optimized for its optimal conditions and position in the sequence of multiplex staining. In order to make the adhesion effect of the tissue better avoid flake baking at 65℃overnight. The pre-staining steps include dewaxing and rehydration, antigen repair, and background quenching.

First step, Four-m sections were deparaffinized using xylene followed by rehydration with different concentrations of ethanol for multiplex immunofluorescence staining .In a second step, antigen retrieval was performed using（1：50 EDTA）with high temperature 30min.

The last step, after cooling to room temperature in a shaker, the slices were soaked in Quenching Buffer and bleached in a special light quenching machine.

Cyclic staining Three successive rounds of antibody staining were performed on each section, each consisting of blocking with 2% normal goat serum in phosphate-buffered saline, incubation with primary antibodies TSA-HRP anti-mouse/rabbit secondary antibodies, fluorophores Cyclic-480,Cyclic-550,Cyclic-630 diluted in TSA diluent, followed by Ab Stripping Buffer. Nuclei were stained with DAPI antibody stripping, the sections mounted with 10% glycerin imaging. Full section scanning was performed on VS200 of Olympus. Following imaging, fluorescence bleaching was repeated in the light quenching machine, excluding Dapi, in preparation for the subsequent round of staining utilizing different indexes. Subsequently, the second round of staining yielded four color staining results.

Control Setup for Multiplex Immunofluorescence Staining:

Single Marker Optimization:

First, stain the same tissue sample with varying concentrations of a single marker antibody to determine the optimal concentration for each marker. This ensures that each marker achieves the best signal strength and specificity.Once the optimal concentration for each marker is determined, proceed with multiplex immunofluorescence staining using these concentrations.

Single Marker Control:

In parallel with the multiplex staining, perform a single marker staining using the same tissue sample and the optimized concentration for each marker. This control is crucial to confirm the specificity and signal intensity of each marker under the conditions of multiplex staining.

Antibody	Species origin	Clone	Dilution	Supplier
CD3	Rabbit	JE80-02	1:500	HUABIO
CD4	Rabbit	ST0488	1:1000	HUABIO
CD8	Mouse	polypeptide	1:300	Immunoway
CD68	Mouse	PDM0-13	1:2000	HUABIO
CD14	Rabbit	SC69-02	1:500	HUABIO
CD20	Rabbit	PD00-02	1:2000	HUABIO
PD-L1	Rabbit	SP142	1:1000	Abcam
PD-1	Mouse	UMAB199	1:800	ZSGB-BIO
Ki67	Rabbit	SR00-02	1:5000	HUABIO

CD31	Mouse	7-A1	1:2000	ThermoFisher
GranzymeB	Rabbit	Polyclonal Antibody	1:2000	HUABIO
α-SMA	Rabbit	SY02-64	1:1000	HUABIO
FAP	Rabbit	R08-9H6	1:500	Zen-BIO
Brg-1	Rabbit	564J7A1	1:1	abcarta

Table S2.Antibody Species origin,clones,dilutions and supplier used for mIF

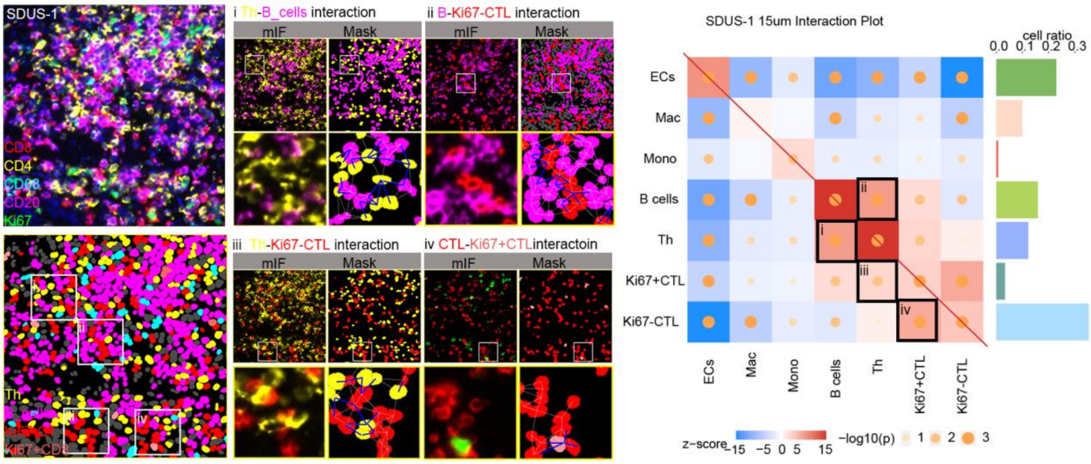

Figure S1 mIF heatmap of cell-cell interactions in patient SDUS-case2, with fluorescence images and rendering of interacting cells.
